# Effects of Er,Cr:YSGG and Diode Lasers on Clinical Parameters and Gingival Crevicular Fluid IL-1*β* and IL-37 Levels in Generalized Aggressive Periodontitis

**DOI:** 10.1155/2019/2780794

**Published:** 2019-06-12

**Authors:** Ahmet Cemil Talmac, Metin Calisir, Emre Gurkan Eroglu, Abdullah Seckin Ertugrul

**Affiliations:** ^1^Yuzuncu Yil University, Faculty of Dentistry, Department of Periodontology, Van, Turkey; ^2^Adiyaman University, Faculty of Dentistry, Department of Periodontology, Adiyaman, Turkey; ^3^Katip Celebi University, Faculty of Dentistry, Department of Periodontology, Izmir, Turkey

## Abstract

**Aim:**

The objective of the current study is to analyze the correlation between cytokine levels and periodontal parameters in aggressive periodontitis patients before and after periodontal treatment that was performed by using two different laser therapies.

**Materials and Methods:**

Twenty-six generalized aggressive periodontitis patients were treated with three different methods (SRP, SRP+diode laser, and SRP+Er,Cr:YSGG laser) applied to three different half-jaws in the same patients. Pre- and posttreatment clinical periodontal parameters and GCF IL-1*β* and IL-37 levels were measured.

**Results:**

There was a statistically significant decrease (*p* < 0.05) between pretreatment and posttreatment clinical periodontal parameters and IL-1*β* and IL-37 levels. When the reduction rates of IL-37 and IL-1*β* levels after treatment were evaluated, the decrease in IL-37 and IL-1*β* levels after treatment was lowest in the SRP group and highest in the SRP+Er,Cr:YSGG group. In addition, the amount of decrease in IL-1*β* in SRP+diode and SRP+Er,Cr:YSGG groups was found to be higher than that in IL-37. Furthermore, there was a positive correlation between IL-37 and IL-1*β* in all groups (*p* < 0.01).

**Conclusion:**

Er,Cr:YSGG laser is more effective than diode laser for the treatment of aggressive periodontitis. IL-37 and IL-1*β* are cytokines that function together and thus must be evaluated together.

## 1. Introduction

Aggressive periodontitis (AgP) is a periodontal disease that is mostly observed in young individuals and characterized by rapidly advancing periodontal tissue destruction and shows genetic predisposition [1]. In a recently published periodontal disease classification criteria, “Classification of Periodontal and Peri-Implant Diseases and Conditions 2017,” aggressive periodontitis was combined with chronic periodontitis to form a single periodontitis category. In this classification, periodontitis characterization is based on the multidimensional staging and grading system [2]. Maintenance of oral hygiene by the patient and scaling and root planing (SRP) processes are the gold standards during treatment [3]. However, since the pathogens have the ability to invade soft tissue, they can persist even after the mechanical treatment. The presence of pathogens in the tissue can reduce the success rate of the treatment and could result in recurrence of the disease [4]. Thus, new approaches are developed for the treatment of aggressive periodontitis, one of which is laser-based therapy.

Use of lasers in periodontology have several advantages such as less pain, less edema, and faster wound healing compared with periodontal surgery. In addition, the laser has bactericidal activity in the application area [5, 6]. Thus, it is advantageous compared to the antibiotic treatment that is performed in addition to the periodontitis treatment, since laser use does not cause bacterial resistance to antibiotics [7]. Therefore, soft tissue lasers such as Erbium, Chromium: Yttrium Scandium Gallium Garnet (Er,Cr:YSGG) laser and diode laser are widely used in different periodontal operations, including the treatment of aggressive periodontitis [6, 8]. The efficacy of Er,Cr:YSGG and diode laser in the treatment of aggressive periodontitis has been previously demonstrated [8–10]. In these studies, one of the parameters to evaluate the treatment success is cytokine levels in the gingival crevicular fluid (GCF) [11, 12], since periodontopathogens and virulence factors result in fast inflammatory and immune responses [13].

The initial response of periodontal tissues to the attack of periodontopathogens is the release of some mediators such as cytokines, kinins, and matrix metalloproteinases (MMPs). This tissue response determines the course of the disease [13, 14]. Cytokines play important roles during the inflammatory response after the tissue destruction and during the initiation, regulation, and continuation of the immune response in periodontal diseases [15]. The cellular responses against proinflammatory cytokines whose effects are restrained by anti-inflammatory cytokines and the equilibrium between these two cytokine types are important in the formation of the inflammatory response [16, 17]. In the GCF samples obtained from the periodontal tissues that showed inflammatory responses, the proinflammatory cytokine levels are higher than the levels in the GCF from the healthy regions [18]. In addition, cytokines are known to have direct and indirect roles in tissue destruction [19, 20]. Therefore, the cytokine response has been suggested to be an important parameter for the pathogenesis of periodontal diseases [21]. Cytokines that are known as innate immunity cytokines such as IL-1, IL-6, and TNF-*α* and IFN-*γ*, IL-4, IL-10, IL-12, IL-17, IL-18, and IL-37 are some of the known proinflammatory and anti-inflammatory cytokines that have been studied in relation to periodontal diseases [22–32].

One of the proinflammatory cytokines causing periodontal tissue destruction is interleukin-1*β* (IL-1*β*). IL-1*β* is an important mediator of the inflammatory response and the pathophysiology of periodontitis and is associated with cell proliferation, differentiation, and apoptosis. It is regarded as a strong gingival crevicular fluid (GCF) biomarker for many parameters, such as severe clinical inflammation, bone destruction, and the progression of periodontal disease. Studies have shown a strong relationship between the severity of periodontal disease and IL-1*β* levels in the gingiva and GCF [33–37]. Another cytokine that is currently widely researched in relation to the inflammatory diseases is IL-37. IL-37, also known as IL-1F7, is one of the 6 new members of the IL-1 family. Although IL-37 is known to function in the inflammation response, its role in different tissues is not fully known [38, 39]. IL-37 was demonstrated to be an anti-inflammatory cytokine consisting of 5 subgroups and acts as a regulatory element during the inflammation response. These findings suggest that IL-37 might be an indicator of several diseases [39–41]. Although IL-37 was recently shown to be associated with inflammatory diseases and could be used as an important parameter in the prognosis of these diseases by reducing proinflammatory cytokine levels [31], the relationship between the expression and function of IL-37 and aggressive periodontitis is limited. Offenbacher et al. [39] reported that the IL-37 variants are associated not only with high inflammatory response but also with more severe clinical findings of the periodontal diseases. IL-37 has also been reported to have broad inhibitory effects on many mediators of the natural immune response, including IL-1*β* [41].

The aim of this study was to determine the efficacy of two different lasers applied in addition to periodontal treatment in generalized aggressive periodontitis patients and to investigate their effects on GCF cytokine levels before and after treatment.

## 2. Materials and Methods

### 2.1. Study Population

A total of 30 subjects, who were treated at the Yuzuncu Yil University Faculty of Dentistry, Department of Periodontology Clinics in 2014-2015, were enrolled in this study; however, 4 patients were excluded due to poor oral hygiene and the lack of compliance with the recommendations. All procedures were approved by the Human Ethics Research Committee of Yuzuncu Yil University (no. B.30.2.YYU.0.01.00.00/44-100912). Diagnoses were made after their clinical and radiographic examinations. It was ensured that the individuals who were included in the study did not have any systemic diseases; were not menopausal, pregnant, or lactating; had not used antibiotics or any other medication affecting the immune system in the previous six months; were nonsmokers; and had not received any periodontal treatment in the previous 6 months. All subjects were informed about the aim and content of the study by a clinician and signed an informed consent form stating that they voluntarily participated in the survey. Each individual read the Helsinki Declaration before joining the study.

### 2.2. Criteria for the Diagnosis of Generalized Aggressive Periodontitis

26 patients (12 female and 14 male) aged between 18 and 35 years (average 31.23 ± 7.4 years), with a clinical diagnosis of generalized aggressive periodontitis, were selected for the study. The selected patients had a minimum of 16 teeth with at least one tooth in each posterior sextant and at least one posterior sextant with a minimum of three natural teeth. The subjects also presented with ≥5 mm of attachment loss around at least seven teeth involved, excluding first molars and central incisors. Patients who had body mass indexes (BMI) that were higher than 25 were not included in the study. BMI values of the patients were between 18.5 and 24.5. According to “Classification of Periodontal and Peri-Implant Diseases and Conditions 2017” (Caton 2018), our patients can be included in the “Stage III and IV, Generalized, Grade C” group, based on the clinical findings, the age of onset, and the clinical course of the disease.

### 2.3. Clinical Measurements

Measurement of clinical parameters was performed by a calibrated clinician. Plaque index (PI) [42], gingival index (GI) [42], bleeding on probing (BOP), probing pocket depth (PPD) (mm), and clinical attachment level (CAL) (mm) were measured at six sites per tooth (mesiobuccal, buccal, distobuccal, distolingual/distopalatinal, lingual/palatinal, and mesiolingual/mesiopalatinal) in all teeth, excluding third molars. BOP was recorded as present or absent if there were signs of bleeding within 30 s after PPD and CAL measurements. Subsequently, the PPD and CAL measurements were recorded to the nearest millimeter using a North Carolina periodontal probe (Hu-Friedy, Chicago, IL, USA). The cementoenamel junction was detected by probing the cervical area of each tooth and was used to calculate the CAL.

### 2.4. Calibration of the Examiner

Calibration of the examiner was performed through a calibration exercise where the examiner measured one quadrant per subject among a group of 10 nonstudy subjects with periodontitis. Each chosen quadrant contained at least six teeth. The examiner measured PPD and CAL in the same quadrant twice with 60 min between measurements, where the same patient was probed twice during the same visit. The variability between measurements was assessed in order to analyze the intraexaminer variability, and calibration was performed through a protocol described previously ^[^43^]^. The standard error of measurement was calculated to be 0.11 mm for PPD and 0.21 mm for CAL measurements.

### 2.5. Study Design

The study was designed as a “split-mouth” study, and all individuals received nonsurgical initial periodontal treatment. At the baseline of the study, all quadrants were shown the similarity of periodontal disease. All participants were treated with the same procedure described below, and three half jaws were randomly selected for the treatment.

1. Only SRP group (SRP-control)
2. SRP+Er,Cr:YSGG laser group (SRP+Er,Cr:YSGG)
3. SRP+940±15 nm diode laser group (SRP+diode)

### 2.6. The Sampling of the Gingival Crevicular Fluid

Before collection of the GCF samples, the supragingival plaque around the probing site was removed and the area was cleaned of saliva through sterile cotton rolls and buffer, after which the tissue was dried with air blowing. The GCF samples were collected from the deepest pocket that was identified after probing measurements. The samples were obtained through paper strips (PerioPaper, Oraflow, NY, USA) both at the beginning of the study and at 3 months after treatment. The uniformly cut paper strips were introduced into the tissue until a light resistance was felt in the sulcus and were kept in the area for 30 seconds. The strips that had any blood on them were excluded from the evaluation. The amount of fluid on the strips was measured by using a Periotron device (Periotron 8000, Oraflow, NY, USA), and the GCF volume was calculated by using these values. Four strips were obtained from each patient and were put into individual 1.5 mL tubes containing 500 *μ*L of phosphate-buffered saline at pH 7.4 and were kept at -80°C.

### 2.7. Er,Cr:YSGG Laser and Diode Laser Application

SRP was first performed to the control quadrants of the individuals diagnosed with aggressive periodontitis, and following the SRP procedure, the Er,Cr:YSGG laser (Waterlase, Biolase, Irvine, CA, USA) was applied. Unlike the Er,Cr:YSGG laser, the 940 ± 15 nm diode laser (iLase, Biolase, Irvine, CA, USA) was applied prior in order to SRP to prevent changing the diode laser efficiency following bleeding. For the Er,Cr:YSGG laser, a 14 mm Z-6 tip (600 *μ*m fiberoptic tip, suitable for periodontal use) marked to the depth of the pocket was used at a setting of 10 Hz, 1.5 W (150 mJ), 65% air, 55% water with H mode, and 140 *μ*s pulse length. The total irradiation time was 30 s. The 940 ± 15 nm diode laser with MZ6-14 mm standard tip was used at a setting in continuous wave mode. The irradiation duration for 940 ± 15 nm diode laser was adjusted to 20 s (Table 1).

### 2.8. ELISA Measurements of IL-37 and IL-1*β* Levels

The IL-37 and IL-1*β* analysis in the GCF was conducted through the ELISA method by using commercial ELISA kits, Human IL-37 ELISA Kit (Hangzhou Eastbiopharm Co. Ltd, Hangzhou, China), and Human IL-1*β* ELISA Kit (AssayMax Human ELISA Kit, Assaypro, Missouri, USA). The evaluation of IL-37 and IL-1*β* was carried out according to the manufacturers' instructions.

Tehe test samples were placed into the wells of the ELISA plate with 100 *μ*L of the standard solutions. The plate was incubated at room temperature for 1 h and was washed 4 times. Afterwards, IL-37 and IL-1*β* detection antibodies were added to each well at a volume of 100 *μ*L. The plate was incubated at room temperature for 30 min and washed 4 times. After adding 100 *μ*L of the color-reactive agent to each well, the plate was incubated at room temperature for 30 min and the reaction was stopped by adding 100 *μ*L of stop solution to each well. The plate was read at 450 nm wavelength by using a microplate reader (Microplate Reader BioTek, VT, USA). GCF IL-37 and IL-1*β* concentrations (pg/mL) were calculated by using the dilution ratio (500 *μ*L) divided by the GCF volume.

### 2.9. Data Analysis

The statistical analysis was carried out using the SPSS 16 package program (SPSS Inc., Chicago, IL, USA). Descriptive statistics, such as the arithmetic means and standard deviation values, were used during the presentation and evaluation of clinical and laboratory data. The comparisons of the pre- and posttreatment values within the group were realized by the Wilcoxon test. The statistical significance of the results was assessed in a 95% reliability interval at the level of *p* < 0.05. A log transformation was performed to normalize the data, and Pearson correlation was done to compare the GCF IL-37 and IL-1*β* levels with a significance set at *p* < 0.01.

## 3. Results

### 3.1. Clinical Findings

26 patients with GAgP, of whom 12 are female and 14 are males, aged 31.23 ± 7.4 years and with no systemic diseases were included in our study. Clinical periodontal indices for all of the individuals included in the study and mean values and standard deviation values regarding pretreatment (day 0) and posttreatment (3rd month) are given in Table 2. According to the results of the statistical evaluation, a decrease of the PI, GI, PPD, CAL, and BOP mean values was observed after the treatment in all three groups (*p* < 0.05) (Table 2).

### 3.2. Cytokine Levels

**Figure 1:**
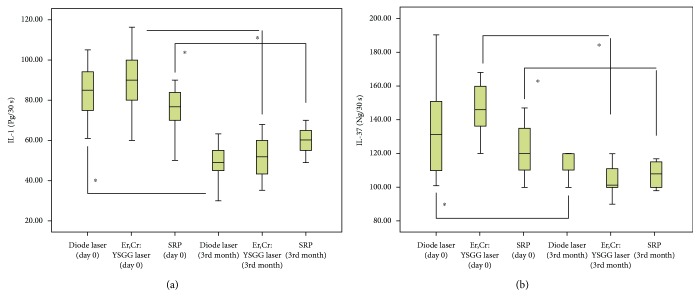
Comparison of cytokine levels at the baseline (day 0) and after the treatment (3rd month) between all groups.^∗^Statistically different from day 0 (for each group) (*p* < 0.05).

**Figure 2:**
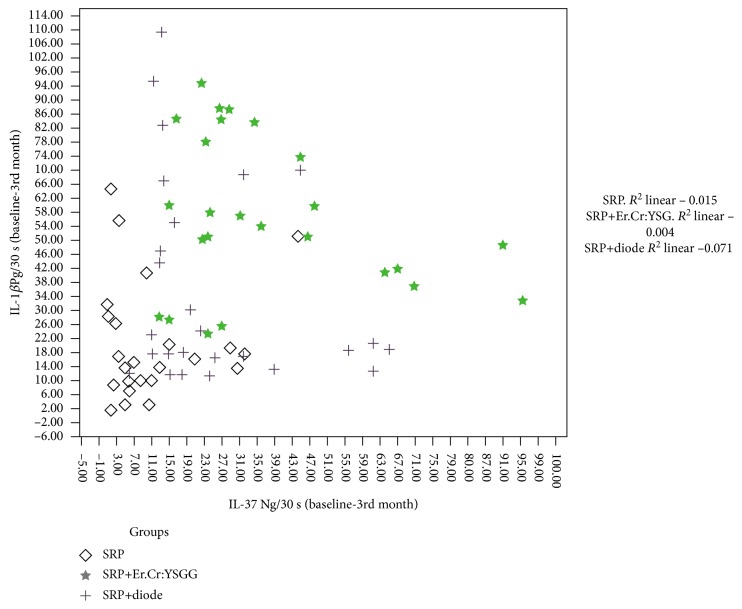
Correlation between the IL-37 (baseline-3rd month) and IL-1*β* (baseline-3rd month) levels in the gingival crevicular fluid of generalized aggressive periodontitis patients. Generalized aggressive periodontitis patients were treated with three different methods (SRP, SRP+Er,Cr:YSGG, and SRP+diode). The relationship between variables was evaluated using the Pearson correlation test (*p* < 0.01).

When IL-1*β* and IL-37 levels in GCF were evaluated, there was a statistically significant decrease in cytokine levels after treatment in all three groups (*p* < 0.05) (Figure 1) (Table 3). In the SRP group, the level of decrease in IL-1*β* concentration was in the range of 2-45 Pg/30 s between the baseline and 3rd month after treatment. In the SRP+diode group, the decrease level of IL-1*β* was in the range of 10-100 Pg/30 s, whereas in the SRP+Er,Cr:YSGG group, the decrease level of IL-1*β* was in the range of 22-98 Pg/30 s (Figure 2). On the other hand, the reduction level of IL-37 in the SRP group was concentrated in the range of 2-35 Ng/30 s. In the SRP+diode group, the reduction level of IL-37 was observed in the range of 7-67 Ng/30 s, whereas in the SRP+Er,Cr:YSGG group, the reduction level of IL-37 was observed in the range of 11-95 Ng/30 s (Figure 2).

Overall, after 3 months, the reduction rate of the IL-1*β* level was higher compared to the reduction rate of the IL-37 level in the SRP+Er,Cr:YSGG group. In addition, there was a weak positive correlation between the reduction rate of IL-1*β* and that of the IL-37 level. In the SRP group, the decrease in the IL-1*β* level at 3 months after periodontal treatment was enhanced compared to the IL-37 level. In addition, there was a strong positive correlation between the reduction rates of IL-1*β* and IL-37 levels. In the SRP+diode group, the decrease rate of the IL-1*β* level was again higher compared to the reduction rate of the IL-37 level at 3 months after periodontal treatment. In addition, there was a strong negative correlation between the reduction rates of IL-1*β* and IL-37 levels after treatment (Figure 2).

## 4. Discussions

The present study analyzes the clinical periodontal parameters and the IL-1*β* and IL-37 levels in the GCF in generalized aggressive periodontitis patients before and after treatment with SRP and with or without additional diode or Er,Cr:YSGG laser therapy. To the best of our knowledge, this is the first study reporting the levels of GCF IL-37 after SRP, SRP+diode laser, and SRP+Er,Cr:YSGG laser therapy in generalized aggressive periodontitis patients.

Different lasers have been used in combination with periodontal therapy [5, 44], such as the Er,Cr:YSGG and diode lasers used in this study. It has been reported that laser treatment reduces the number of periodontopathogens by focusing on the biofilm layer, removing tartar and bacterial toxins from the root surface, decreasing pocket depth by removing the sulcular epithelium and inducing the formation of new connecting epithelia, and stimulating wound healing promoting healing [45]. There are many studies on the use of diode laser in periodontology in the literature [9, 46–48]. Controlled clinical trials evaluating the treatment of moderate and deep pockets in patients with aggressive periodontitis have shown that the diode laser application in addition to SRP improves the clinical outcome of the periodontal treatment [9, 49, 50]. Although several studies have investigated the efficacy of Er,Cr:YSGG laser use in periodontology [10, 51–54], there are very few controlled clinical studies analyzing the efficacy of Er,Cr:YSGG laser in aggressive periodontitis patients [8, 55]. According to a study investigating the effects of Er,Cr:YSGG laser in patients with chronic and aggressive periodontitis, the use of this laser in addition to periodontal treatment led to a significant decrease in the number of periodontal pathogens and thus helped to maintain periodontal health [55]. Similarly, our study showed that the application of both of these lasers in addition to SRP resulted in an improvement of the clinical parameters and GCF cytokine levels.

The number of studies that have compared the efficacy of these lasers when they are used in combination with periodontal therapy is limited. In a study evaluating the effects of Er,Cr:YSGG and diode laser in aggressive and chronic periodontitis patients, Ertugrul et al. found that both lasers decreased the human *β*-defensin and IL-1*β* levels more than the SRP-alone treatment group. They also reported that Er,Cr:YSGG laser is more effective in the treatment of generalized aggressive periodontitis and chronic periodontitis compared to diode laser [8]. Similar to this study, we found that diode and Er,Cr:YSGG laser application was beneficial on the clinical periodontal parameters and GCF cytokine levels when used in addition to the classical periodontal treatment.

Cytokines play an important role in the pathogenesis of periodontal diseases [14, 56, 57]. Inflammatory cytokines are induced throughout the inflammatory response in periodontal diseases and are closely related to the onset and/or progression of periodontal disease [37, 58]. The cytokine levels were similar in the inflamed periodontal tissues of individuals with both chronic periodontitis and aggressive periodontitis. On the other hand, T-cell levels were higher and macrophage counts were lower in aggressive periodontitis patients compared to chronic periodontitis patients. However, it is very difficult to compare the cytokine responses between these diseases, because, as the disease progresses from an early stage to a more advanced stage, it is possible that there would be temporary changes in the cytokine profiles. Since only the chronic stages of chronic periodontitis are evaluated, it is possible that there is a change in the cellular or cytokine profiles of the early stages of the disease. In addition, other issues, including genetic diversity, the presence or absence of certain microorganisms, and the severity and duration of the disease, may also affect cellular populations [59, 60].

Proinflammatory cytokines increase inflammation and osteolysis in the periodontal tissues, while anti-inflammatory cytokines repress the synthesis of proinflammatory cytokines preventing or at least slowing the tissue destruction [61]. Some of these cytokines are present in the GCF and are used as disease indicators [62]. In the present study, we analyzed the levels of IL-1*β*, and an anti-inflammatory cytokine IL-37 in the GCF samples of periodontitis patients before and after treatment, and their association with other clinical parameters.

IL-1 is a multifactorial cytokine which exhibits strong inflammatory properties and has the ability to activate many cell types. IL-1 is mostly released by macrophages in addition to monocytes [63]. IL-1 is a polypeptide that is involved in the tissue destruction and hemostasis [64] and has local and systemic effects on immune and inflammatory systems [64, 65]. While IL-1 has 11 subtypes [64], IL-1*β* is the most common form [66]. Although it has similar biological properties with IL-1*α*, IL-1*β* expression is 10-50 times more than IL-1*α* and it is much more potent [67, 68]. In their study evaluating the efficacy of Er,Cr:YSGG and diode lasers in aggressive and chronic periodontitis patients, Ertugrul et al.[8] reported that both lasers decrease the levels of GCF IL-1*β*. Similarly, in our study, a significant reduction in the level of GCF IL-1*β* was observed after treatment with both lasers.

IL-37 is the newest member of the IL-1 cytokine family. Although most of the 11 members of the IL-1 family are proinflammatory, IL-37 is anti-inflammatory [69]. IL-37 can be upregulated by proinflammatory cytokines, and its levels increase during the disease progression and decrease during healing [70]. IL-37 has 5 isoforms found in different tissues. In one study, it was found that the IL-37b (isoform1) is the dominant IL-37 isoform in the human gingival tissue. In the same study, it was also found that IL-37b levels were significantly higher in gingival tissues with periodontitis, and immunohistochemistry experiments have shown that IL-37 expression and localization was increased in gingival tissue with periodontitis, especially in the infiltrate in the connective tissue and epithelium [41]. Similarly, in our study, GCF IL-37 levels in samples from inflamed areas in aggressive periodontitis patients were found to be high before treatment, while a significant decrease was observed in the level of GCF IL-37 after treatment.

IL-37 is usually released in inflamed tissues [71]. The presence of cytokines such as IL-1*β*, IL-18, TNF-*α*, IFN-*γ*, and TGF-*β* can increase the synthesis of IL-37 in inflammatory diseases [72], which in turn suppresses the release of proinflammatory cytokines such as IL-1*β*, IL-16, IL-18, and TNF-*α* [31, 41]. These results clearly show the anti-inflammatory properties of IL-37. In the present study, GCF IL-1*β* and IL-37 cytokine levels were also found to be increased during inflammation. IL-37 probably increased due to elevated IL-1*β* cytokine levels in the inflamed periodontal tissues prior to aggressive periodontitis treatment. After the treatment, however, we observed a reduction in IL-37 levels for all 3 treatment groups, likely due to the clinical healing of periodontal tissues and the decrease of elevated proinflammatory cytokine levels.

There are many studies on the possible roles of IL-37 in inflammatory response; however, there are very few studies examining the role of IL-37 in the area of periodontology. Sağlam and colleagues **[**73**]** analyzed the correlation between the levels of IL-37 in the GCF, saliva, and blood with clinical periodontal parameters. The study consisted of 20 periodontally healthy subjects, 20 gingivitis patients, and 20 chronic periodontitis patients. IL-37 was reported to be present in the GCF, saliva, and blood; thus, this biomarker is not exclusive to periodontal tissues. In comparison among groups, it was suggested that IL-37 levels were not correlated with the GCF volume. In addition, there was no correlation between the clinical periodontal parameters and IL-37 levels in the GCF, saliva, and blood. These results suggested that IL-37 is not an effective parameter for the diagnosis of or for determining the progression of periodontal disease. In contrast to these results, our results suggest that IL-37 levels correlate with clinical periodontal parameters and IL-1*β* levels. In all three groups, a statistically significant decrease was observed in the posttreatment levels of IL-1*β* and IL-37 compared to their levels before treatment. The decrease in the levels of IL-37 in addition to the levels of other cytokines (IL-1*β*) in association with the clinical parameters was found to be statistically significant. We propose that there is a correlation between IL-37 and IL-1*β* levels and periodontal disease, and levels of IL-37 and IL-1*β* correlation may be an important parameter to evaluate the efficiency of the treatment of aggressive periodontal disease.

## 5. Conclusion

The levels of proinflammatory cytokine IL-1*β*, which plays a role in the pathogenesis of aggressive periodontitis, were shown to be positively correlated with the IL-37 levels. These data indicate that IL-37 may play an important role in protecting periodontal tissues from excessive inflammatory response. Therefore, IL-37 may be used as a novel treatment biomarker for generalized aggressive periodontitis diagnosis. However, further studies are needed to elucidate the regulatory mechanisms of IL-37 in the pathogenesis of generalized aggressive periodontitis.

## Figures and Tables

**Table 1:** Diode laser and Er,Cr:YSGG laser specifications.

	Diode laser	Er,Cr:YSGG laser
Type	MZ6-14 mm	14 mm Z-6
Irradiation times	20 s	30 s
Duration of treatment session	2	2
Energy density	13.5 J/cm^2^	15 J/cm^2^
Laser wavelength	940 ± 15 nm	2780 *μ*m
Methods	10 Hz, 1.5 W (150 mJ), 65% air, 55% water with H mode, 140 *μ*s pulse length	1.5 W with a pulse interval of 20 ms and pulse length of 20 ms delivering 20 s/cm^2^

**Table 2:** Comparison of mean values and standard deviations of clinical periodontal indices at the baseline (day 0) and after the treatment (3rd month).

Diode laser			Er,Cr:YSGG laser		SRP (control)		*p* values
	Day 0	3rd month	Day 0	3rd month	Day 0	3rd month	—
PI (M ± SD)	1.54 ± 0.19	1.23 ± 0.2^∗^	1.53 ± 0.25	1.13 ± 0.1^∗^	1.51 ± 0.2	1.26 ± 0.1^∗^	<0.05
GI (M ± SD)	1.52 ± 0.22	1.22 ± 0.2^∗^	1.53 ± 0.23	1.15 ± 0.1^∗^	1.46 ± 0.19	1.35 ± 0.2^∗^	<0.05
PPD (mm) (M ± SD)	3.99 ± 0.76	3.28 ± 0.6^∗^	3.96 ± 0.59	3.15 ± 0.4^∗^	3.91 ± 0.6	3.34 ± 0.4^∗^	<0.05
CAL (mm) (M ± SD)	4.4 ± 0.76	3.69 ± 0.6^∗^	4.56 ± 1.04	3.71 ± 0.7^∗^	4.5 ± 0.93	3.9 ± 0.86^∗^	<0.05
BOP% (M ± SD)	51.92 ± 19.9	26.92 ± 19^∗^	53.84 ± 23.12	15.38 ± 15^∗^	50 ± 18.7	28.84 ± 13^∗^	<0.05

PI: plaque index, GI: gingival index, PPD: probing pocket depth, CAL: clinical attachment level, BOP: bleeding on probing.^∗^Statistically different from day 0 (for each group) (*p* < 0.05).

**Table 3:** Comparison of mean values and standard deviations of cytokine levels at the baseline (day 0) and after the treatment (3rd month).

Diode laser			Er,Cr:YSGG laser		SRP (control)		Variation between day 0 and 3rd month (*p* values)
	Day 0	3rd month	Day 0	3rd month	Day 0	3rd month	
IL-1*β* (M ± SD) Pg/30 s	84.20 ± 43.60	50.69 ± 33^∗^	92.61 ± 56.15	53.7 ± 38^∗^	77.5 ± 46.15	57.9 ± 41^∗^	<0.05
IL-37 (M ± SD) Ng/30 s	131.66 ± 34.71	109.36 ± 3^∗^	136.42 ± 34.33	100.6 ± 27.1^∗^	125.43 ± 35.03	114.5 ± 33^∗^	<0.05

∗Statistically different from day 0 (for each group) (*p* < 0.05).

## Data Availability

All the generated or analysed data used to support the findings of this study are included within the article.
